# Effects of Trandolapril on Structural, Contractile and Electrophysiological Remodeling in Experimental Volume Overload Heart Failure

**DOI:** 10.3389/fphar.2021.729568

**Published:** 2021-09-10

**Authors:** Dagmar Jarkovská, Matúš Miklovič, Jitka Švíglerová, Luděk Červenka, Petra Škaroupková, Vojtěch Melenovský, Milan Štengl

**Affiliations:** ^1^ Department of Physiology, Faculty of Medicine in Pilsen, Charles University, Pilsen, Czechia; ^2^ Biomedical Center, Faculty of Medicine in Pilsen, Charles University, Pilsen, Czechia; ^3^ Center for Experimental Medicine, Institute for Clinical and Experimental Medicine, Prague, Czechia; ^4^ Department of Pathophysiology, 2^nd^ Faculty of Medicine, Charles University, Prague, Czechia; ^5^ Department of Cardiology, Institute for Clinical and Experimental Medicine, Prague, Czechia

**Keywords:** cardiac remodeling, volume overload, aortocaval fistula, rat, renin-angiotensin-aldosterone system, trandolapril

## Abstract

Chronic volume overload induces multiple cardiac remodeling processes that finally result in eccentric cardiac hypertrophy and heart failure. We have hypothesized that chronic angiotensin-converting enzyme (ACE) inhibition by trandolapril might affect various remodeling processes differentially, thus allowing their dissociation. Cardiac remodeling due to chronic volume overload and the effects of trandolapril were investigated in rats with an aortocaval fistula (ACF rats). The aortocaval shunt was created using a needle technique and progression of cardiac remodeling to heart failure was followed for 24 weeks. In ACF rats, pronounced eccentric cardiac hypertrophy and contractile and proarrhythmic electrical remodeling were associated with increased mortality. Trandolapril substantially reduced the electrical proarrhythmic remodeling and mortality, whereas the effect on cardiac hypertrophy was less pronounced and significant eccentric hypertrophy was preserved. Effective suppression of electrical proarrhythmic remodeling and mortality but not hypertrophy indicates that the beneficial therapeutic effects of ACE inhibitor trandolapril in volume overload heart failure might be dissociated from pure antihypertrophic effects.

## Introduction

Heart failure (HF) is a complex clinical syndrome characterized by cardiac dysfunction, i.e., the inability to maintain sufficient cardiac output, and myocardial structural abnormalities including hypertrophy and dilated cardiomyopathy. It is considered an epidemic disease in the modern world, affecting approximately 1–2% of the adult population and its prevalence is increasing (Savarese and Lund, 2017). HF is a multifactorial and systemic disease; the most common etiologies are ischemic heart disease, hypertension, and diabetes (Lloyd-Jones et al., 2002). HF activates a variety of structural, neurohumoral, cellular, and molecular mechanisms, which compensate for the decrease in mean arterial pressure due to reduced cardiac output (Kemp and Conte, 2012). These compensatory responses comprise the Frank-Starling Law, mainly in the early stages of HF (Westerhof and O’Rourke, 1995), stimulation of the sympathetic nervous system with release of catecholamines (Chaggar et al., 2009), activation of the renin-angiotensin-aldosterone-system (RAAS) (Ruzicka et al., 1993; Ruzicka et al., 1995; Rea and Dunlap, 2008), ventricular remodeling (Schirone et al., 2017), and the release of many neurohormones with vasoactive effects (Kim and Januzzi, 2011). Despite the early beneficial effects, long-term sympathetic and RAAS activations result in maladaptive ventricular remodeling with deleterious effects on cardiac function and accelerated progression of HF (Lee and Tkacs, 2008; Triposkiadis et al., 2009). The significant role of RAAS in HF progression is also supported by a number of beneficial effects of drugs affecting this signaling pathway such as ACE inhibitors, angiotensin receptor blockers and mineralocorticoid receptor antagonists (Orsborne et al., 2017).

Rats with chronic volume overload due to aortocaval fistula (ACF rats) represent a well-characterized, reproducible and simple model of heart failure (Abassi et al., 2011). This model was adapted from dogs to rats in the early 1970s (Stumpe et al., 1973; Hatt et al., 1979; Hatt et al., 1980) and simplified by the needle procedure in the early 1990s (Garcia and Diebold, 1990). The model reproduces several characteristic features of human heart failure, e.g. gradual development from asymptomatic into decompensated phase, cardiac hypertrophy and dilatation as well as myocardial remodeling (Liu et al., 1991a; Liu et al., 1991b), elevated cardiac filling pressures, diminished effective cardiac output (despite elevated total cardiac output in ACF rats), neurohumoral activation and altered calcium handling (Flaim et al., 1979; Lee and Tkacs, 2008; Abassi et al., 2011). Special features distinct from those of pressure overload and infarction models include a lack of fibrosis and inflammation as well as the activation of different signaling pathways (Ruzicka et al., 1994; Toischer et al., 2010). Cardiac hypertrophy is often associated with increased susceptibility to ventricular arrhythmias and sudden cardiac death due to electrical remodeling with action potential prolongation, altered electrotonic coupling among the cells, slower conduction, and dispersion of refractoriness (Hill, 2003).

In this study we have hypothesized that multiple remodeling processes due to volume overload might be affected differentially by chronic ACE inhibition possibly allowing their dissociation. The objectives were to describe volume overload-induced structural, contractile and electrophysiological cardiac remodeling and to analyze the effects of chronic trandolapril administration on remodeling processes as well as on overall mortality.

## Materials and Methods

Animal handling was in accordance with the European Directive for the Protection of Vertebrate Animals Used for Experimental and Other Scientific Purposes (86/609/EU). The experiments were approved by the Committee for Experiments on Animals of the Charles University Faculty of Medicine in Pilsen and by the Ministry of Education, Youth and Sports of the Czech Republic (Protocol No. MZ-5809/2015-OZV-30.0-5.2.15). In this study, 62 Hannover Sprague-Dawley male rats (8 weeks old) were used. The animals were divided into three groups: sham-operated rats (sham surgery and placebo treatment), ACF rats (aortocaval fistula and placebo treatment), and ACF rats with trandolapril (aortocaval fistula and trandolapril treatment). The drug treatment started 4 weeks after the surgery and continued for 20 weeks. Experimental time points of 4 weeks (start of trandolapril treatment) and 24 weeks (*in vivo* and *in vitro* analyses) after surgery were chosen based on previous studies and our experience with the model. The time point of 4 weeks after surgery represents the compensatory stage with eccentric dilation, moderately impaired pump function and minimal mortality (Brower et al., 1996; Brower and Janicki, 2001; Ryan et al., 2007), in which reverse cardiac remodeling can be induced by therapeutic intervention (Hutchinson et al., 2011; Brower et al., 2015). The time point of 24 weeks after surgery was chosen as the end point, at which overt congestive heart failure should be developed in most animals (Brower and Janicki, 2001) and the mortality still allows a systematic analysis of cardiac function (Oliver-Dussault et al., 2010; Melenovsky et al., 2012). Trandolapril was administered in drinking water, 6 mg/L (Gopten; Abbot, Prague, Czech Republic). The dosing was based on previous studies (Červenka et al., 2015a; Červenka et al., 2015b), in which the effective suppression of angiotensin II plasma and tissue levels was demonstrated. In the placebo groups, the animals were given tap water.

### Surgery

Prior to the surgery the animals were anaesthetized using ketamine and midazolam applied intraperitoneally (Calypsol, Gedeon Richter, Hungary, 160 mg/kg and Dormicum, Roche, France, 160 mg/kg). The aortocaval fistula was created as described previously (Garcia and Diebold, 1990). The abdominal aorta was pierced into the inferior vena cava by an 18-gauge needle (1.2 mm in diameter). A bulldog vascular clamp was placed across the caudal aorta, the needle was removed, and an acrylamide tissue glue (Histoacryl, B.Braun AG, Germany) was applied at the puncture point. After 30 s, the clamp was removed and the aortocaval shunt was checked visually by vena cava pulsation. The sham-operated rats underwent only opening and closing of the abdominal cavity, without the aortocaval fistula procedure. As post-operative analgesia, meloxicam (1–2 mg/kg/day SC) was given for 2–3 days.

### *In Vivo* Experiments

Cardiac examination was performed 24 weeks after the surgery on anaesthetized animals (50 mg/kg of ketamine and 5 mg/kg of midazolam i.p.). Electrocardiography (ECG) was measured as previously (Sedmera et al., 2016) (sham-operated rats, *n* = 13; ACF rats, *n* = 10; and ACF rats with trandolapril, *n* = 9). In brief, four ECG leads were recorded using FE 132 Bioamp and Powerlab 8 (ADInstuments, Ltd., Australia) at a sampling rate of 1 kHz. Offline analyses of 200 consecutive beats in the lead with the least noise from each recording was performed in LabChart Pro 7 (ADInstruments, Ltd.). The beats chosen by the software classifier as good ones were averaged and the program measured the RR intervals, PR intervals, QRS intervals, and QT intervals automatically. The QTc intervals were determined using Bazett’s formula normalized to the average RR interval [QTc = QT/(RR/f)^1/2^, f = 160 ms], (Kmecova and Klimas, 2010).

Echocardiography (sham-operated rats, *n* = 15; ACF rats; *n* = 15, and ACF rats with trandolapril, *n* = 15) was performed with a 10 MHz probe (Vivid System 5, GE, United States). The aortic blood pressure (sham-operated rats, *n* = 13; ACF rats, *n* = 14; and ACF rats with trandolapril, *n* = 14) was measured using Powerlab 8 (ADInstruments Ltd.) and a 2F micro-manometer catheter (Millar Instruments, TX, United States) put into the aorta and left ventricle via the carotid artery. The offline analysis was made in LabChart Pro 7 (ADInstruments Ltd.) (Melenovsky et al., 2018).

### *In Vitro* Experiments

The animals were sacrificed by cervical dislocation 24 weeks after the surgery. The hearts were excised, weighed, and used either for multicellular (sham-operated rats, *n* = 12; ACF rats, *n* = 10; and ACF rats with trandolapril, *n* = 10) or for cellular (8 cells/3 rats in each group) experiments. The multicellular experiments included measurements of atrial electrical activity in isolated atria and of contraction force and membrane potential in ventricular trabeculae from the left ventricles. The spontaneous atrial activity was recorded in a warm oxygenated (37°C) experimental chamber filled with Tyrode solution (in mmol/L: NaCl 137, KCl 4.5, MgCl_2_ 1, CaCl_2_ 2, glucose 10, and HEPES 5; pH was adjusted to 7.4 with NaOH; all chemicals were purchased from Sigma-Aldrich, Czech Republic) using Biopac System (Biopac Systems Inc., Santa Barbara, CA, United States). After a 5-min stabilization period, norepinephrine was added to the bath solution to increase the concentration every 3 min (10^−8^, 10^−7^, 10^−6^, 10^−5^, and 10^−4^ mol/L). Analysis of the acquired signal was performed offline using Biopac Student Lab PRO 3.7.2 (Biopac Systems Inc., Santa Barbara, CA, United States). Values of the heart rate obtained from this analysis were normalized to the heart rate in the control solution (Tyrode solution without norepinephrine).The ventricular preparations (trabeculae isolated from the left ventricle) were placed into a measurement chamber perfused with oxygenated warm (37°C) Tyrode solution (in mmol/L: NaCl 137, KCl 4.5, MgCl_2_ 1, CaCl_2_ 2, glucose 10, and HEPES 5; the pH was adjusted to 7.4 with NaOH; all chemicals were purchased from Sigma-Aldrich, Czech Republic) and stimulated at various frequencies (0.5, 1, 2, 3, and 5 Hz; Pulsemaster Multi-Channel Stimulator A300, World Precision Instruments, Inc., FL, United States). Contraction force was measured by an isometric force transducer (model F30; Hugo Sachs Electronik, Harvard Apparatus, GmBH, Germany) and membrane potential was measured using glass microelectrodes filled with 3M KCl (resistance >20 MΩ; Microelectrode Puller P-1000, Sutter Instruments, CA, United States). Action potential duration at 75% repolarization and maximal contraction force were measured offline in 10 consecutive beats by in-house software made in MATLAB 2014b (MathWorks Inc., MA, United States). These results were averaged and used for statistical analysis.

The ventricular cardiomyocytes were isolated by enzymatic dissociation. After excision of the heart, the aorta was cannulated and the heart was mounted to a constant-pressure Langendorff apparatus, where it was perfused with warm (37°C) oxygenated solution: 1) 3 min Ca^2+^-free Tyrode solution; 2) 12 min 0.5 mmol/L Ca^2+^ Tyrode solution with collagenase A (1 g/L, Sigma-Aldrich) and bovine serum albumin (0.5 g/L, Sigma-Aldrich); 3) 3 min Ca^2+^-free Tyrode solution. The digested ventricular tissue was cut into cubic pieces (1 mm^3^), mechanically agitated and the debris filtered. The Ca^2+^ concentration in the solution with cardiomyocytes was gradually (1 µmol/L, 5 µmol/L, 10 µmol/L, 0.1 mmol/L) increased up to 0.2 mmol/L. Calcium transients and sarcomeric contractions were measured by Ionoptix HyperSwitch Myocyte Calcium and Contractility System (IonOptix LLC, CA, United States) with the Sarclen sarcomere length acquisition module. The cardiomyocytes were incubated in 1 mmol/L Fura-2-AM solution (Molecular Probes, Invitrogen, MA, United States) dissolved in dimethyl sulfoxide (Sigma-Aldrich) for 20 min in 2 mmol/L Ca^2+^ Tyrode solution. After the incubation, the cells were washed with 2 mmol/L Ca^2+^ Tyrode solution repeatedly. During the measurement, the cardiomyocytes were placed in an experimental chamber perfused with warm (37°C) 2 mmol/L Ca^2+^ Tyrode solution and stimulated with the MyoPacer Field Stimulator (IonOptix LLC, CA, United States) at various frequencies (0.5, 1, 2, 3, and 5 Hz). Offline analysis of the calcium transients and the sarcomeric length was realized in IonWizard 6.5 (IonOptix LLC, CA, United States).

### Statistical Analysis

The results of the experiments (presented as mean ± SD) were checked for normality distribution using the Shapiro-Wilk test and outliers were excluded by the Grubbs test. The boxplots show mean (dot), median (horizontal line), SD (box) and max-min (whiskers). The comparisons were made with a one-way ANOVA test followed by a post hoc Tukey test to determine possible significant differences between the experimental groups. The two-way mixed-design ANOVA (with stimulation frequency as a repeated-measures factor and experimental groups as a non-repeated-measures factor), followed by a post hoc Tukey test was used to analyze the results of *in vitro* experiments. Survival analysis was performed with the Kaplan-Meier estimator. The incidence of spontaneous activity in ventricular cardiomyocytes was compared by Fisher’s exact test between experimental groups; *p* < 0.05 was considered statistically significant. The whole statistical analysis described above was performed in OriginPro 2019 (OriginLab Corporation, Northampton, MA, United States).

## Results

**FIGURE 1:**
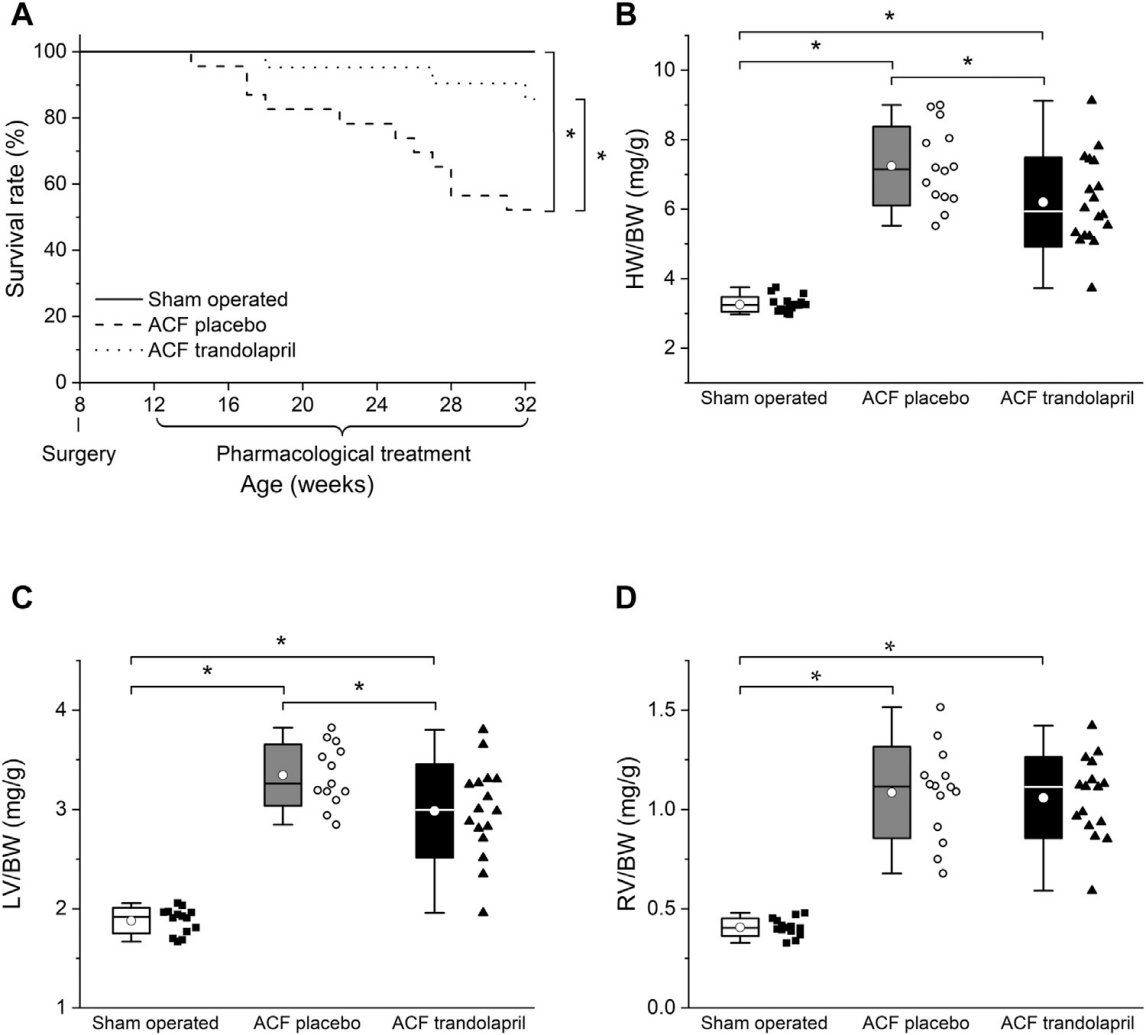
Survival and heart weights. **(A)** Survival rates in sham-operated rats (*n* = 18), ACF rats (*n* = 23) and ACF rats with trandolaprl (*n* = 21). **(B)** Heart weight to body weight ratios in sham-operated rats (*n* = 12), ACF rats (*n* = 10) and ACF rats with trandolapril (*n* = 10). **(C)** Left ventricle weight to body weight ratios in sham-operated rats (*n* = 12), ACF rats (*n* = 10) and ACF rats with trandolapril (*n* = 10). **(D)** Right ventricle weight to body weight ratios in sham-operated rats (*n* = 12), ACF rats (*n* = 10) and ACF rats with trandolapril (*n* = 10). *p < 0.05.

In ACF rats, the administration of trandolapril significantly improved the survival rate (Figure 1A) to values similar to sham-operated animals (24 weeks after surgery survived 100% of sham-operated animals, 52% of ACF rats, and 86% of ACF rats with trandolapril). Chronic volume overload in ACF rats induced the development of cardiac hypertrophy in both left and right ventricles (Figures 1B–D). The hypertrophy was slightly attenuated by trandolapril in the left ventricle (Figure 1C; left ventricle weight to body weight ratio of 1.88 ± 0.13 mg/g in sham-operated rats, of 3.35 ± 0.31 mg/g in ACF rats, and of 2.98 ± 0.47 mg/g in ACF rats with trandolapril), but not in the right ventricle (Figure 1D; right ventricle weight to body weight ratio of 0.41 ± 0.04 mg/g in sham-operated rats, of 1.09 ± 0.23 mg/g in ACF rats, and of 1.06 ± 0.21 mg/g in ACF rats with trandolapril).

**FIGURE 2:**
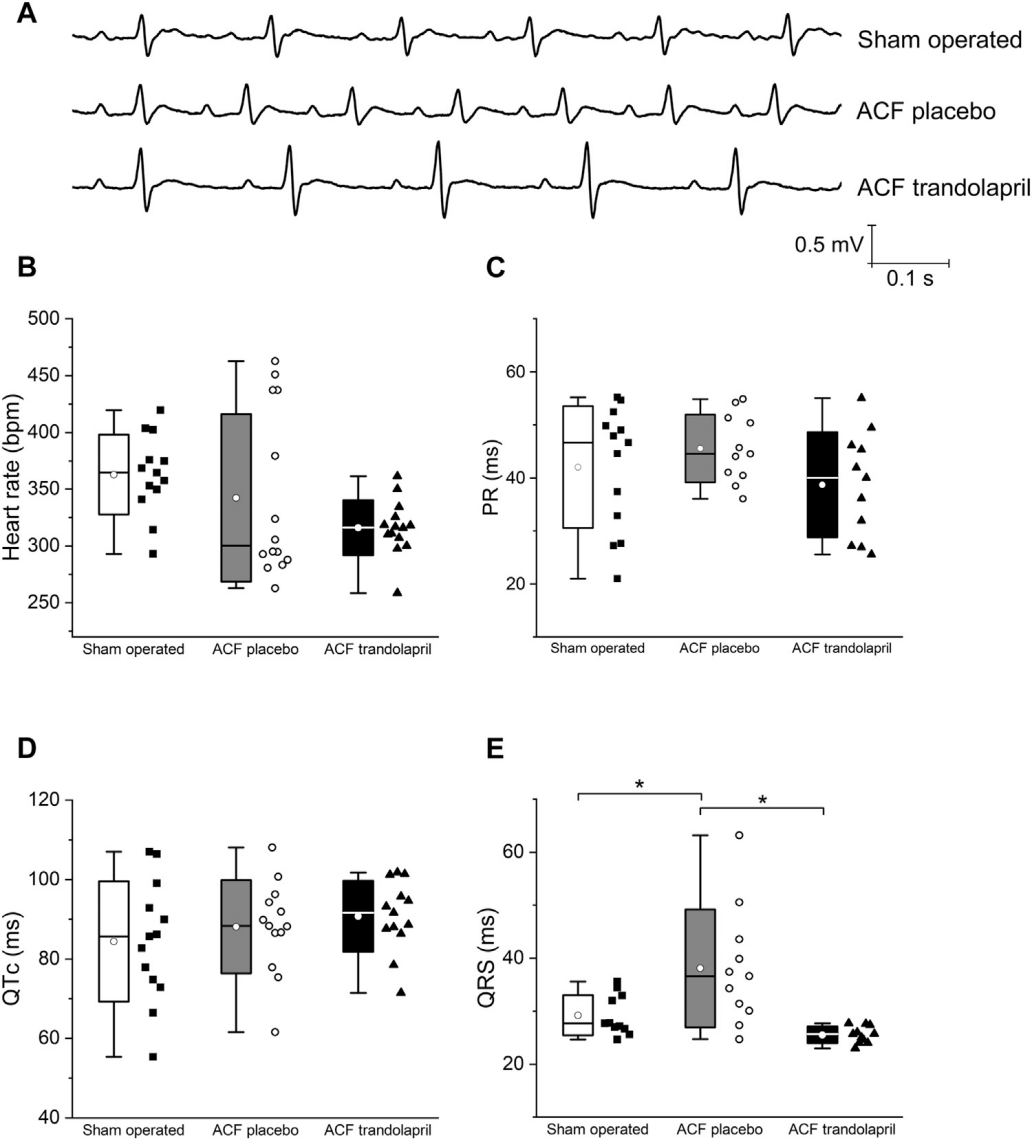
Electrocardiographic intervals. **(A)** Representative ECG tracings in a sham-operated rat, an ACF rat, and an ACF rat with trandolapril. **(B)** Heart rates in sham-operated rats (*n* = 13), ACF rats (*n* = 10) and ACF rats with trandolapril (*n* = 9). **(C)** PR intervals in sham-operated rats (*n* = 13), ACF rats (*n* = 10) and ACF rats with trandolapril (*n* = 9). **(D)** QTc intervals in sham-operated rats (*n* = 13), ACF rats (*n* = 10) and ACF rats with trandolapril (*n* = 9). **(E)** QRS intervals in sham-operated rats (*n* = 13), ACF rats (*n* = 10) and ACF rats with trandolapril (*n* = 9). *p < 0.05.

Electrocardiographic analysis revealed similar heart rates, PR and QTc intervals in all three experimental groups (Figures 2A–D). The QRS interval was significantly prolonged in ACF rats; this effect was suppressed by the administration of trandolapril (Figure 2E; QRS interval of 29 ± 4 ms in sham-operated rats, of 38 ± 11 ms in ACF rats, and of 26 ± 2 ms in ACF rats with trandolapril).

**FIGURE 3:**
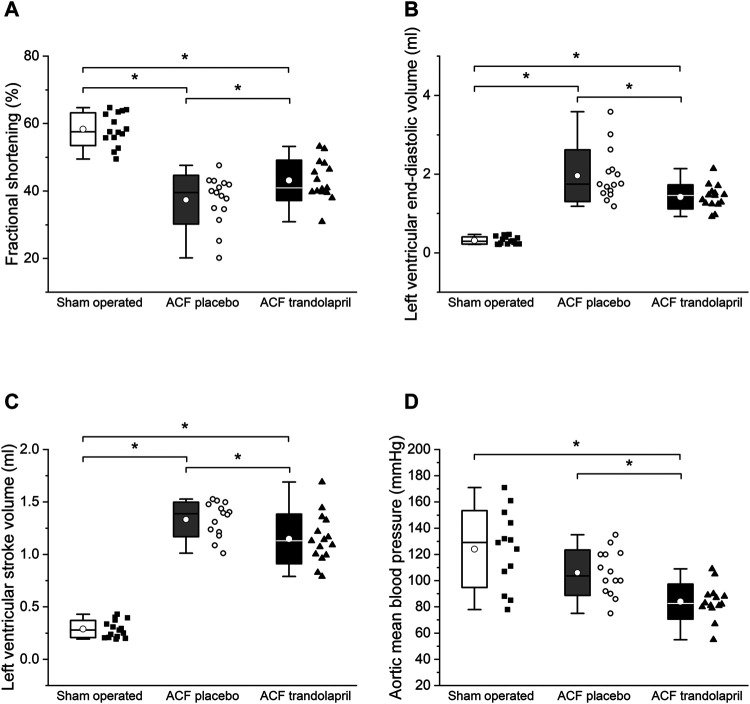
Hemodynamics by echocardiography. **(A)** Left ventricular fractional shortening in sham-operated rats (*n* = 15), ACF rats (*n* = 15) and ACF rats with trandolapril (*n* = 15). **(B)** Left ventricular end-diastolic volume in sham-operated rats (*n* = 15), ACF rats (*n* = 15) and ACF rats with trandolapril (*n* = 15). **(C)** Left ventricular stroke volume in sham-operated rats (*n* = 15), ACF rats (*n* = 15) and ACF rats with trandolapril (*n* = 15). **(D)** Mean arterial (aortic) blood pressure in sham-operated rats (*n* = 13), ACF rats (*n* = 14) and ACF rats with trandolapril (*n* = 14). *p < 0.05.

Echocardiographic measurements showed a significant reduction of left ventricular fractional shortening in ACF rats, which was partially reversed by trandolapril (Figure 3A). The left ventricular diameters and volumes were markedly increased in ACF rats, but significantly less in ACF rats treated with trandolapril (Figures 3B,C; Table 1). Left ventricular posterior wall and septal thickness was decreased in ACF rats and trandolapril did not affect it (Table 1). Systemic vascular resistance was substantially reduced in ACF rats and remained low in the presence of trandolapril (Table 1). Cardiac output was increased in ACF rats regardless of trandolapril treatment (Table 1). Mean arterial (aortic) blood pressure was decreased only in ACF rats treated with trandolapril (Figure 3D), due to a decrease in both systolic and diastolic blood pressures (Table 1).The lung/body weight ratio was increased in ACF rats as well as in ACF rats with trandolapril, whereas the liver/body weight ratio was not influenced by either intervention (Table 1).

**TABLE 1:** Echocardiography.

Parameter	Sham operated	ACF placebo	ACF trandolapril
End-diastolic left ventricular internal diameter (mm)	6.73 ± 0.65	12.39 ± 1.29*	11.19 ± 0.8*^,#^
End-systolic left ventricular internal diameter (mm)	2.81 ± 0.51	7.82 ± 1.67*	6.38 ± 0.93*^,#^
End-diastolic interventricular septal thickness (mm)	2.41 ± 0.24	1.84 ± 0.18*	1.86 ± 0.28*
Left ventricular posterior wall thickness (mm)	2.68 ± 0.29	2.13 ± 0.22*	1.93 ± 0.22*
Systemic vascular resistance (dyn·s/cm^5^)	1730 ± 426	349 ± 76*	290 ± 72*
Left ventricular cardiac output/body weight (mL/min/g)	0.23 ± 0.05	0.83 ± 0.11*	0.83 ± 0.15*
Aortic systolic blood pressure (mmHg)	138 ± 31	130 ± 22	102 ± 13*^,#^
Aortic diastolic blood pressure (mmHg)	109 ± 28	83 ± 15*	66 ± 13*
Lung weight/body weight (mg/g)	4.13 ± 1.14	5.18 ± 1.00*	5.51 ± 1.21*
Liver weight/body weight (mg/g)	35.7 ± 5.3	29.5 ± 3.8	30.6 ± 8.1

*Significantly different from sham operated, *p* < 0.05.

^#^Significantly different from ACF placebo, *p* < 0.05.

**FIGURE 4:**
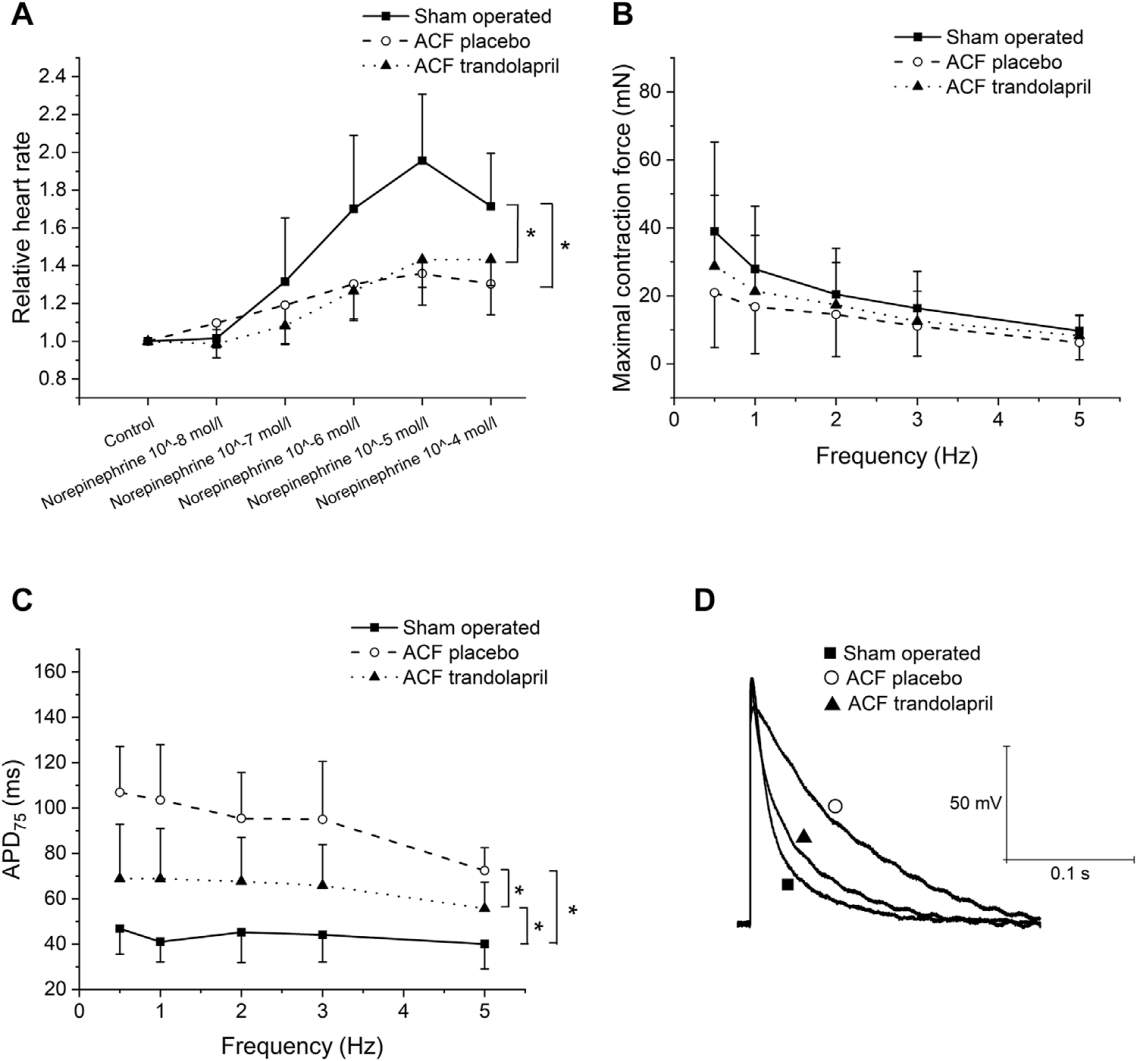
Isolated cardiac tissue. **(A)** Effect of norepinephrine on spontaneous activity of isolated atria from sham-operated rats (*n* = 12), ACF rats (*n* = 10) and ACF rats with trandolapril (*n* = 10). **(B)** Contraction force in left ventricle trabeculae of sham-operated rats (*n* = 12), ACF rats (*n* = 10) and ACF rats with trandolapril (*n* = 10). **(C)** Action potential duration at 75% repolarization (APD_75_) in left ventricle trabeculae of sham-operated rats (*n* = 12), ACF rats (*n* = 10) and ACF rats with trandolapril (*n* = 10). **(D)** Representative cellular action potentials in left ventricle trabeculae of a sham-operated rat, an ACF rat, and an ACF rat with trandolapril at stimulation frequency of 1 Hz. *p < 0.05.

In isolated atria, the norepinephrine-induced increase in heart rate was significantly reduced in ACF rats, and treatment with trandolapril did not affect it (Figure 4A; heart rate increased by 96 ± 35% in sham-operated rats, by 36 ± 17% in ACF rats, and by 43 ± 15% in ACF rats with trandolapril at norepinephrine concentration of 10^−5^ mol/L). In left ventricle isolated trabeculae, the contraction force was similar in all three groups regardless of stimulation frequency (Figure 4B). Action potential duration was significantly prolonged in ACF rats at all stimulation frequencies (1–5 Hz) and treatment with trandolapril suppressed the prolongation (Figures 4C,D; APD_75_ of 47 ± 11 ms in sham-operated rats, of 107 ± 20 ms in ACF rats, and of 69 ± 24 ms in ACF rats with trandolapril at stimulation frequency of 0.5 Hz). Similar results were obtained also for right ventricle papillary muscles (not shown).

**FIGURE 5:**
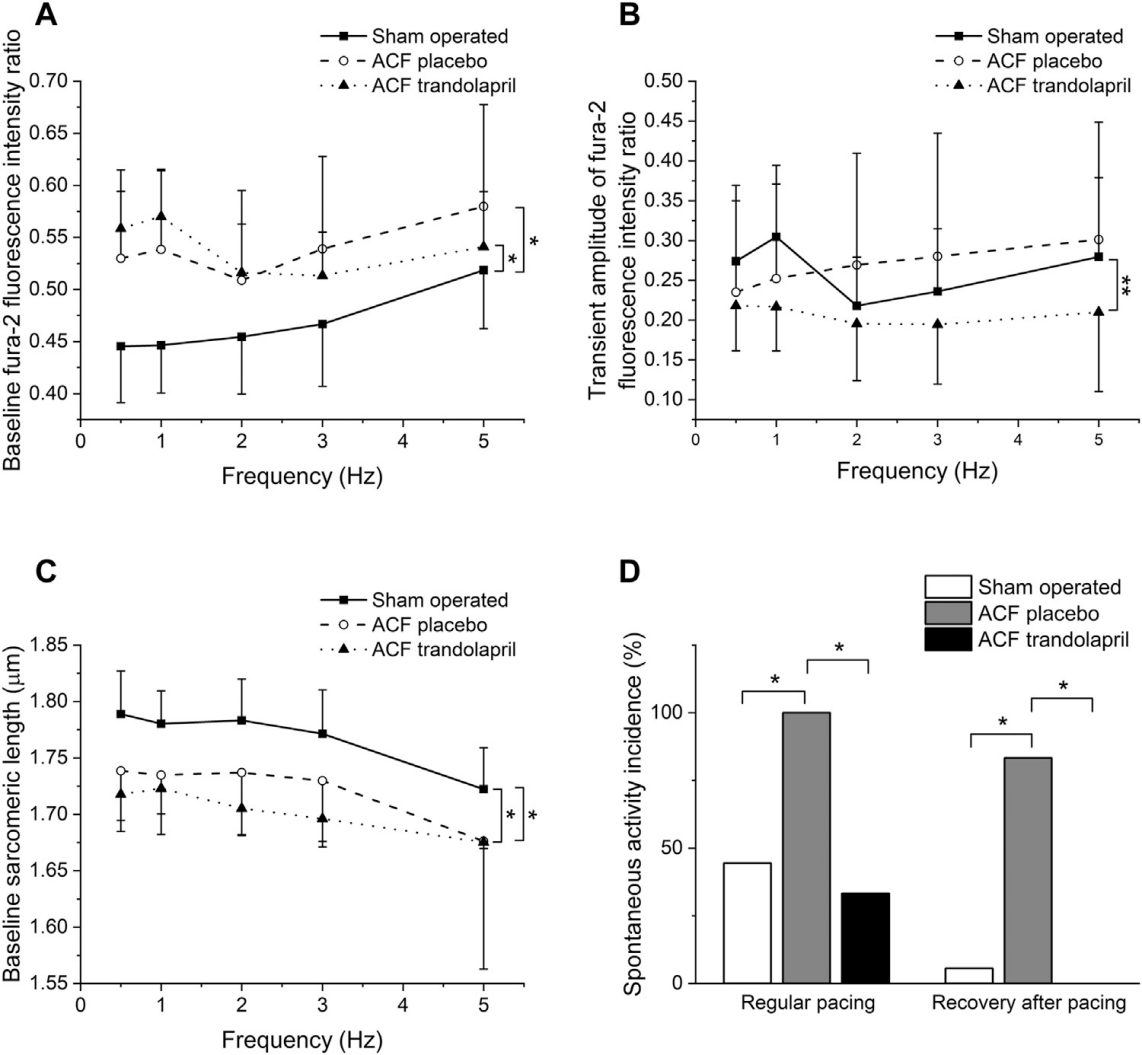
Intracellular Ca^2+^ and sarcomeric length in isolated ventricular myocytes. **(A)** Baseline Fura-2 fluorescence ratios in isolated ventricular myocytes from sham-operated rats (*n* = 8), ACF rats (*n* = 8) and ACF rats with trandolapril (*n* = 8). **(B)** Transient amplitudes of Fura-2 fluorescence ratios in isolated ventricular myocytes from sham-operated rats (*n* = 8), ACF rats (*n* = 8) and ACF rats with trandolapril (*n* = 8). **(C)** Baseline sarcomeric length in isolated ventricular myocytes from sham-operated rats (*n* = 8), ACF rats (*n* = 8) and ACF rats with trandolapril (*n* = 8). **(D)** Incidence of spontaneous activity in isolated ventricular myocytes from sham-operated rats, ACF rats and ACF rats with trandolapril during regular pacing and during recovery after fast (5 Hz) pacing. *p < 0.05.

Baseline Fura-2 fluorescence ratios that reflect intracellular Ca^2+^ concentration in isolated ventricular myocytes at rest were higher in cells from ACF rats, both treated and untreated with trandolapril (Figure 5A; baseline fluorescence ratio of 0.45 ± 0.05 in sham-operated rats, of 0.53 ± 0.06 in ACF rats, and of 0.56 ± 0.06 ms in ACF rats with trandolapril at stimulation frequency of 0.5 Hz). Ca^2+^ transient amplitudes of Fura-2 fluorescence ratios were not significantly different (Figure 5B). The intracellular Ca^2+^ levels corresponded well with sarcomeric lengths, which were at baseline the highest in myocytes from sham-operated rats (Figure 5C; baseline sarcomeric length of 1.79 ± 0.04 µm in sham-operated rats, of 1.74 ± 0.05 µm in ACF rats, and of 1.72 ± 0.02 µm in ACF rats with trandolapril at stimulation frequency of 0.5 Hz) and the contraction shortenings were similar in all experimental groups (not shown). In isolated ventricular myocytes, spontaneous activity was observed. The incidence of spontaneous activity was increased in myocytes from ACF rats and this increase was suppressed by trandolapril treatment, both during period of regular pacing (spontaneous activity developed in 8 out of 18 sham myocytes, in 12 out of 12 ACF myocytes and in 4 out of 12 ACF+trandolapril myocytes) and period of recovery after fast pacing (spontaneous activity developed in 1 out of 18 sham myocytes, in 10 out of 12 ACF myocytes and in 0 out of 12 ACF+trandolapril myocytes; Figure 5D).

## Discussion

Chronic volume overload due to aortocaval fistula led to pronounced cardiac hypertrophy (both left and right ventricles) and increased mortality. Trandolapril substantially reduced the mortality; the effect on cardiac hypertrophy, however, was less pronounced and significant hypertrophy was preserved in ACF rats with trandolapril treatment. Echocardiography revealed decreased fractional shortening in ACF rats, which was only moderately and incompletely reversed by trandolapril. *Ex vivo* experiments in isolated cardiac tissues (trabeculae) and ventricular myocytes did not show any significant contractile remodeling, similar to a previous study (Ryan et al., 2007). Several proarrhythmic electrophysiological alterations that developed in ACF rats, including the widening of the QRS complex, prolongation of APD, and increased spontaneous activity of cardiac myocytes, were suppressed by trandolapril treatment. All in all, chronic volume overload was associated with pronounced structural, contractile, and electrophysiological cardiac remodeling, which probably contributed to the increased mortality in ACF rats. Effective suppression of electrophysiological proarrhythmic changes, as well as of mortality, by trandolapril might suggest a significant contribution of arrhythmic deaths to the overall mortality of ACF rats.

In heart failure, defective Ca^2+^ homeostasis, together with electrical remodeling, contributes to enhanced susceptibility to cardiac arrhythmias and arrhythmia-induced sudden death (Luo and Anderson, 2013). Impaired Ca^2+^ handling in failing hearts due to altered expression and/or function of multiple proteins and signaling pathways results in decreased Ca^2+^ transients and elevated diastolic intracellular Ca^2+^ levels (Lou et al., 2012). Electrical remodeling involves changes in various ionic currents leading to prolongation of action potential duration and potential arrhythmogenesis (Cutler et al., 2011). The electrical remodeling and Ca^2+^ homeostasis are closely interconnected through a number of Ca^2+^-dependent signaling pathways (e.g. Ca^2+^- and calmodulin-dependent protein kinase II, CaMKII), with intracellular Ca^2+^ emerging as a central player in maladaptive remodeling and arrhythmogenesis (Luo and Anderson, 2013). In ACF rats, diastolic (baseline) intracellular Ca^2+^ levels were increased and action potentials were significantly prolonged. These alterations could contribute to proarrhythmic substrate and/or trigger, as evidenced by increased spontaneous activity in isolated ventricular myocytes. Increased susceptibility to ventricular arrhythmias in ACF rats with a high frequency of ventricular ectopic beats and bursts of ventricular tachycardia degenerating into lethal ventricular fibrillation was also demonstrated in an earlier study (Benes et al., 2011). Since trandolapril suppressed both the manifestations of proarrhythmic substrate (action potential duration prolongation, spontaneous activity, wide QRS complex) and mortality, it is tempting to speculate that arrhythmic deaths contribute significantly to overall mortality and that trandolapril might prevent them through inhibiting proarrhythmic remodeling.

The aortocaval shunt led to a pronounced reduction of total systemic vascular resistance and an increased cardiac output as reported earlier (Liu et al., 1991a; Liu et al., 1991b). The increased cardiac output was due to an increase in stroke volume, the heart rate was not affected. The aortic systolic blood pressure was not changed in ACF rats, whereas the aortic diastolic blood pressure was reduced, again consistent with previous report (Liu et al., 1991b). Increased dimensions of left ventricle, both end-systolic and end-diastolic, with slightly reduced wall thickness, document development of an eccentric hypertrophy in ACF rats. Collectively, these findings support the concept that the chronic volume overload triggers, through increased wall stress and neurohumoral activation, compensatory biventricular hypertrophy, which eventually deteriorates into heart failure (Kehat and Molkentin, 2010). The hemodynamic load, however, is different for the right and left ventricles: whereas in the left ventricle pure volume overload is present, the right ventricle faces a mixed pressure and volume overload, leading to a distinct pattern of activation of cardiac growth factors (Modesti et al., 2004). Differential mechanisms of the left *vs.* right ventricular hypertrophy are also supported by the selective antihypertrophic effect of trandolapril in the left ventricle only (this study). The different sensitivity of ventricles to the antihypertrophic effect of trandolapril might be due to different signaling pathways activated in either ventricle (Modesti et al., 2004) and/or to the differential effects of trandolapril on systemic and pulmonary circulation. In agreement with the latter hypothesis, in a clinical study chronic trandolapril administration was found to reduce mean arterial systemic blood pressure (as shown in our study), but not to influence pulmonary artery pressure (van der Ent et al., 1998).

Multiple beneficial effects of trandolapril have been demonstrated. The antihypertensive effects are based on decreased levels of angiotensin II, catecholamines, and vascular remodeling. In patients with left ventricular systolic dysfunction due to myocardial infarction, trandolapril decreased the mortality rate, incidence of atrial fibrillation, risk of sudden death, and development of severe heart failure (Diaz and Ducharme, 2008). Similar to clinical studies, multiple vascular and cardiac effects of trandolapril were also demonstrated in animal models (Fornes et al., 1992; Koffi et al., 1998; Tanonaka et al., 2001). The relationships between various levels of cardiac remodeling and trandolapril effects, however, remain unclear. In healthy guinea pig isolated hearts, electrophysiological effects of ACE inhibition were shown; the effects, however, were not substantial enough to produce either antiarrhythmic or proarrhythmic effects (Gilat et al., 1998). In middle-aged spontaneously hypertensive rats, the relationships between structural and electrical remodeling and trandolapril treatment were investigated (Chevalier et al., 1995). The structural remodeling was defined in terms of hypertrophy and fibrosis, with the electrical proarrhythmic remodeling as prevalence of premature ventricular beats. Based on a correspondence analysis, a strong correlation between hypertrophy, fibrosis, and ventricular premature beats was found only for severe hypertrophy; for moderate hypertrophy, the correlation disappeared. This indicates that the electrical remodeling is not always linked to structural remodeling, and additional causal factors (e.g., Ca^2+^ homeostasis) must be taken into account. The results obtained in ACF rats support this view of rather independent structural (hypertrophy) and functional (electrical proarrhythmic) remodeling (at least in the early stages of heart failure), although the hypertrophic phenotype of ACF rats did not include excessive fibrosis (Benes et al., 2011). Whereas the hypertrophy was preserved (only slightly affected) in ACF rats treated with trandolapril, the electrical proarrhythmic remodeling and mortality were almost completely suppressed. It should be realized, however, that only surviving rats were analyzed in tissue and cellular experiments and therefore the most advanced stages of heart failure (i.e., non-survivors) probably were not included. In a study on the long-term outcome of the volume overload rat model, 28% of the animals were found to die without previous heart failure signs, probably due to arrhythmia-related death (Melenovsky et al., 2012).

Besides direct cardiac effects, the vascular effects of trandolapril could also contribute to better ventricular-vascular coupling and improved cardiac function. Reduction of elevated blood pressure by ACE inhibitors including trandolapril was reported to decrease arterial stiffness and pulse wave velocity (Topouchian et al., 1999; Ichihara et al., 2003; Meani et al., 2018), which was associated with a significant regression of cardiac hypertrophy. Nevertheless, preserved cardiac hypertrophy and the absence of hypertension in our model rather argue against this mechanism, although the slight decrease in LV/BW ratio might be attributed to the aortic pressure difference between ACF rats and ACF rats with trandolapril.

After initial beneficial effects of sympathetic stimulation, the chronic activation of adrenergic signaling pathways in heart failure leads to multiple detrimental changes that decrease responsiveness to the adrenergic signaling and impair Ca^2+^ handling (Lou et al., 2012). This was manifested in our study by a significantly lower norepinephrine-induced heart rate elevation *in vitro* in the ACF groups. Similarly, in a porcine volume-overload ACF model the decreased heart rate responsiveness to adrenergic stimulation was described *in vivo* together with increased norepinephrine plasma levels, decreased myocardial norepinephrine content, number of β1-adrenergic receptors, cAMP production, and cardiac G_s_-protein (Hammond et al., 1992). In ACF rats, elevated norepinephrine plasma levels with depletion of cardiac norepinephrine stores due to sympathetic denervation were described (Willenbrock et al., 1997; Kristen et al., 2006). An inverse relation between the abundance of sympathetic marker tyrosin hydroxylase and left ventricular hypertrophy severity and/or degree of congestion also suggests a blunted β-adrenergic signaling in this model (Sedmera et al., 2016).

An analysis of interactions of volume overload and of trandolapril on several levels of biological complexity showed some apparent discrepancies. Although an *in vivo* electrocardiogram did not show any significant differences in QT/QTc intervals between the experimental groups, the action potential durations were markedly prolonged in ACF rats. The absence of differences in cardiac repolarization *in vivo* was probably due to relatively high heart rates and complex regulatory mechanisms present *in vivo*. Removal of these superimposed control mechanisms unmasked the intrinsic repolarization differences that reflect a lower repolarization reserve in the hearts of ACF rats and consequently a higher propensity of cardiac myocytes to triggered activity (Varró and Baczkó, 2011). Furthermore, a significant *in vivo* prolongation of QTc interval in ACF rats was shown in a recent study (Wang et al., 2019), in which, however, the duration of volume overload period was shorter (12 weeks *vs.* 24 weeks in our study) and anesthesia was different (chloral hydrate *vs.* ketamine/midazolam in our study) compared to our study. The wide QRS complexes indicate impaired spreading of cardiac excitation in ACF rats, probably due to connexin 43 downregulation and hypophosphorylation (Sedmera et al., 2016). Reduction of echocardiographic fractional shortening in ACF rats was not translated to a corresponding decrease in the contraction force of multicellular preparations or sarcomeric shortening of isolated myocytes. This discrepancy could be related to changes in chamber geometry, differential preload/afterload conditions, and again to the absence of *in vivo* regulatory mechanisms in cellular experiments. Blunted sympathetic regulation (Willenbrock et al., 1997; Kristen et al., 2006; Sedmera et al., 2016) could contribute to the reduced *in vivo* fractional shortening. Furthermore, cellular remodeling might be heterogeneous with transmural, regional, and interventricular differences, as suggested by some discrepant results of our and previous studies (Ryan et al., 2007; Guggilam et al., 2013).

From a clinical point of view, it should be emphasized that volume overload represents a proarrhythmic condition. In a large cohort of patients with isolated mitral valve prolapse, ventricular arrhythmias were frequent although rarely severe. Nevertheless, for arrhythmic mitral valve prolapse, long-term severe arrhythmia was independently associated with notable excess mortality and reduced event-free survival (Essayagh et al., 2020). Similarly, in a porcine model of pulmonary regurgitation and volume overload, increased incidence of inducible ventricular and atrial arrhythmias was found (Zeltser et al., 2005). Our study indicates that the proarrhythmic remodeling and arrhythmic event risk associated with volume overload can be reduced by angiotensin-converting enzyme inhibitors regardless of their anti-hypertrophic effects. To the best of our knowledge, such a combination of several proarrhythmic factors in volume overload and their regression by ACE inhibitors, together with a reduction of mortality despite preserved hypertrophy, was never shown; this sheds light on the unclear beneficial effects of ACE inhibitors in conditions of volume-overload heart failure. In general, the beneficial effects of ACE inhibitors in heart failure were associated with antihypertrophic effects and attenuation of ventricle enlargement in a number of both experimental and clinical studies (Pfeffer et al., 1992; St John Sutton et al., 1994; Brower et al., 2015). However, in pure volume overload conditions, the effects of ACE inhibitors remain unclear: Although, in rats with aortic regurgitation, ACE inhibitors reduced left ventricle hypertrophy and improved survival (Arsenault et al., 2013), they failed to attenuate left ventricle remodeling in dogs with mitral regurgitation (Dell’italia et al., 1997; Perry et al., 2002) and rats with aortocaval fistula (Ryan et al., 2007). Inconsistent results have also been obtained in patients with aortic insufficiency (Mahajerin et al., 2007). In this context, the evidence of reduced mortality and regression of proarrhythmic electrophysiological remodeling despite the preservation of substantial hypertrophy is novel and suggests the dissociation of antiarrhythmic and antihypertrophic effects of ACE inhibitors in volume overload.

### Study Limitations

Although several manifestations of proarrhythmic electrophysiological remodeling were demonstrated in this study, life-threatening ventricular arrhythmias were not documented in our experimental design. Previous studies documented lethal ventricular arrhythmias in ACF rats (Benes et al., 2011) and a significant incidence of arrhythmia-related deaths was suggested (Melenovsky et al., 2012). Telemetric studies that would allow long-term and repeated ECG monitoring, and recording of arrhythmic events will be necessary for understanding mechanisms of arrhythmia initiation and perpetuation in this rat model.

Detailed analyses of both upstream and downstream elements of relevant signaling pathways and remodeling targets were beyond the scope of this study. Proteomic and transcriptomic analysis of hearts from ACF rats revealed the differential expression of 66 myocardial proteins and 851 differentially expressed mRNAs (Petrak et al., 2011), which testifies to the complexity of such a task. Patch-clamp investigations of ionic currents showed a reduction in potassium currents I_K_ and I_K1_ in hypertrophied cardiomyocytes from ACF rats (Alvin Z. V. et al., 2011). L-type calcium current (I_CaL_) was shown to be either decreased (Alvin Z. et al., 2011) or unchanged (Ding et al., 2008). For major calcium transporters, the downregulation of SERCA2 and ryanodine receptor proteins was reported (Ding et al., 2008; Petrak et al., 2011). In another study, an unchanged protein expression of SERCA2 was found, together with reduced protein expression of phospholamban and Na/Ca^2+^ exchanger (Harris et al., 2007). Collectively, these changes in ionic currents and calcium transporters correspond with our findings and might underlie them.

Trandolapril was administered in drinking water. Although the effective suppression of angiotensin II plasma and tissue levels with such administration and dosing was demonstrated (Červenka et al., 2015a; Červenka et al., 2015b), the variable intake of drinking water could contribute to the observed variability of some results.

Only male rats were used for the study, to decrease the gender-related heterogeneity of the results. The higher susceptibility of male hearts to ventricular remodeling induced by chronic volume overload was demonstrated in an earlier study (Gardner et al., 2002).

## Conclusion

Chronic volume overload due to aortocaval fistula induced pronounced structural, contractile, and electrophysiological cardiac remodeling, which resulted in increased mortality. The effective suppression of electrical proarrhythmic remodeling and mortality, but not hypertrophy, indicates that the therapeutic effects of the ACE inhibitor trandolapril in volume-overload heart failure might be dissociated from the purely antihypertrophic effects.

## Data Availability

The raw data supporting the conclusions of this article will be made available by the authors, without undue reservation.
